# 

**Published:** 1845-08-01

**Authors:** 


☞ We learn that T. Rush Spencer, M. D., late adjunct Prof. of Mat., Med. and Pathol. in Geneva College, has received the appointment of Professor of Mat. Medica in Willoughby Medical College, Ohio, and has signified his acceptance thereof. Dr. S. has a reputation for talent and acquirement, with a facility for teaching, which promise a career of usefulness and honor in the responsible duties upon which he is about to enter.

